# Systemic immune modulation by stereotactic radiotherapy in early-stage lung cancer

**DOI:** 10.1038/s41698-023-00358-z

**Published:** 2023-03-02

**Authors:** Eleni Gkika, Elke Firat, Sonja Adebahr, Erika Graf, Ilinca Popp, Gianluca Radicioni, Simon S. Lo, Ursula Nestle, Nils H. Nicolay, Gabriele Niedermann, Dan G. Duda, Anca-L. Grosu

**Affiliations:** 1 Department of Radiation Oncology, Medical Center—University of Freiburg, Faculty of Medicine, Freiburg, Germany; 2 German Cancer Consortium (DKTK), Partner Site Freiburg, German Cancer Research Center, Heidelberg, Germany; 3 Institute of Medical Biometry and Statistics, Medical Center—University of Freiburg, Faculty of Medicine, Freiburg, Germany; 4 Department of Radiation Oncology, University of Washington School of Medicine, Seattle, WA USA; 5 Department of Radiation Oncology, Kliniken Maria Hilf, Monchengladbach, Germany; 6 E. L. Steele Laboratories for Tumor Biology, Department of Radiation Oncology, Massachusetts General Hospital and Harvard Medical School, Boston, MA USA

**Keywords:** Non-small-cell lung cancer, Radiotherapy

## Abstract

We performed a prospective study of circulating immune cell changes after stereotactic body radiotherapy (SBRT) in 50 early-stage NSCLC patients. We found no significant increase in CD8^+^ cytotoxic T lymphocytes at first follow-up (the primary endpoint) but detected a significant increase in expanding Ki-67^+^CD8^+^ and Ki-67^+^CD4^+^ T-cell fractions in patients treated with 10 Gy or less per fraction. SBRT can induce significant expansion in circulating effector T-cells immediately post-treatment.

Stereotactic body radiotherapy (SBRT) is an essential treatment modality for early-stage and oligometastatic non-small cell lung cancer (NSCLC)^1,2^. SBRT induces DNA double-strand breaks, leading to cell killing. SBRT may also modulate systemic immunity, which is relevant given the increasing role of immune checkpoint blockade (ICB) in NSCLC. Prior studies suggested that SBRT can lead to increased activated NK lymphocytes and decreased regulatory T cells (Tregs)^3^. However, the immunomodulatory effects of SBRT remain incompletely characterized^4^.

CD8^+^ cytotoxic T lymphocytes (CTLs) can mount responses against many human cancer types^5,6^. However, CTL responses are often insufficient to eradicate tumors^6,7^. SBRT may promote systemic immune activation through pleiotropic effects. For example, the use of non-ablative doses of 3x8Gy can lead to immunogenic death; the resultant anti-tumor immune response has the potential to control non-irradiated lesions^8^. But how to activate anti-tumor CTL responses using SBRT while avoiding RT-induced lymphodepletion and what is the optimal RT dose (ablative versus non-ablative) and fractionation schedule remain outstanding questions. Answering these questions is critical for effectively combining SBRT with ICB.

We prospectively evaluated the impact of ablative single-site SBRT on systemic immunity in early-stage NSCLC patients. We used immunoprofiling of peripheral blood cells by longitudinal assessment at first SBRT fraction (baseline), during and at the end of SBRT, and at first (FU1) and second (FU2) follow-up (1½ and 4½ months after SBRT, respectively). The primary endpoint was an increase (yes/no) in circulating CD8^+^ CTL counts at FU1 versus pre-treatment. Secondary endpoints included changes in other T-cell subsets at all time-points. The study accrued 56 NSCLC patients between 2016–2021, of whom 50 were evaluable (4 dropped out, 2 withdrew consent). Patients and treatment characteristics are shown in Supplementary Table 1.

The absolute counts of circulating CD8^+^ CTLs at FU1 compared to baseline increased only in 21% of the patients (not significant). Moreover, there was a significant decrease in the mean absolute counts of CD8^+^ CTLs and CD4^+^ T-cells at all time-points compared to pre-treatment values (Fig. 1a, b and Supplementary Fig. 1a, b). Although prior studies suggested that SBRT can reduce RT-induced lymphopenia in patients with NSCLC^9^ or pancreatic cancer^10^, our data show significant lymphodepletion during and after ablative SBRT in NSCLC patients, despite the smaller irradiated volume and no nodal irradiation (Supplementary Tables 1 and 3).

**Fig. 1:**
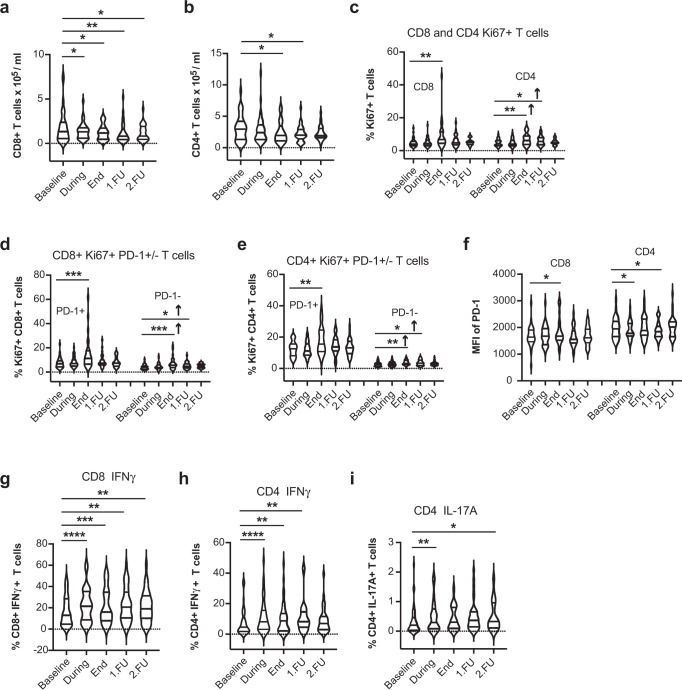
Transient lymphodepletion during SBRT and increased proliferation of CD8^+^ and CD4^+^ circulating T-cells after SBRT in early-stage NSCLC patients. Absolute counts of CD8^+^ (**a**) and CD4^+^ circulating T-cells (**b**). Fractions of Ki-67^+^CD8^+^ and Ki-67^+^CD4^+^ T-cells (**c**), Ki67^+^PD-1^+^/ Ki67^+^PD-1^−^ CD8^+^ T-cells (**d**), and Ki-67^+^PD-1^+^ versus Ki-67^+^PD-1^−^ CD4^+^ T-cells (**e**) during and after SBRT. **f** Median fluorescence intensity (MFI) of PD-1 immunostaining for CD8^+^ and CD4^+^ T cells. Expression of IFN-γ in CD8^+^ T cells (**g**) and CD4^+^ T-cells (**h**), and expression of IL-17A in CD4^+^ T-cells upon re-stimulation (**i**). *p < 0.05, **p < 0.01, ***p ≤ 0.001, ****p ≤ 0.0001 from mixed effects model for repeated measures with Geisser-Greenhouse correction and Benjamini, Krieger, and Yekutieli for the false discovery rate, two-sided. Arrows indicate the direction of change. Data are shown as median values (center lines) and interquartile ranges.

We then sought to examine the changes in Ki-67, a marker of cellular proliferation expressed by cycling or recently divided cells^6,11–13^. Interestingly, the proportion of proliferating CD4^+^ and CD8^+^ T-cells among peripheral blood lymphocytes (CD3^+^ cells) significantly increased after SBRT (end of treatment, *p* = 0.003 and *p* = 0.006, respectively) (Fig. 1c and Supplementary Fig. 1c). These increases occurred in the PD-1^+^ subset, which may include tumor-specific T-cells^14–17^, but also in the PD-1^−^ subset (Fig. 1d, e and Supplementary Fig. 1d, e). Moreover, median fluorescence intensity of PD-1 immunostaining was also higher at the end of treatment in the CD8^+^ and CD4^+^ T-cells, indicative of an increased expression level (Fig. 1f and Supplementary Fig. 1f). Additionally, the fractions of T-cells expressing the activation marker IFN-γ and IL-17A increased during and after SBRT (Fig. 1g–i and Supplementary Fig. 1g–i).

Overall, there was a significant decrease in naïve and memory CD8^+^ and CD4^+^ T-cell subpopulations after SBRT (Supplementary Fig. 2a, b and Supplementary Fig. 3a, b). Nevertheless, the fractions of CD8^+^ and CD4^+^ T-cells expressing inducible costimulatory (ICOS) increased at FU1 (Supplementary Figs. 2c and 3c). Tregs are considered more radioresistant but also significantly decreased after SBRT at FU1 (Supplementary Figs. 2d and 3d). Similarly, myeloid-derived suppressor cells (MDSC)—which promote tumor progression^18^—decreased at post-treatment time-points (Supplementary Figs. 2e and 3e). TIM3 and CTLA-4 expression was detected only on a minority of circulating T-cells, indicating that most circulating T-cells were not terminally exhausted after SBRT (Supplementary Figs. 2f, g and 3f, g). All results are summarized in Supplementary Table 3.

In addition, we performed further sub-group analyses after stratifying for RT dose, using the median dose per fraction (10 Gy) as the cut-off point. NSCLC patients treated with 10 Gy or less (*n* = 25) showed significant increases in the proportion of CD8^+^ and CD4^+^ proliferating T-cells compared to pre-treatment values. In contrast, we detected no changes in patients who received more than 10 Gy per fraction (*n* = 19) (Fig. 2a and Supplementary Fig. 4a). The same changes were seen in the proliferating PD1^+^ or PD1^−^ CD8^+^ and CD4^+^ T-cell fractions (Fig. 2b, c and Supplementary Fig. 4b, c). The choice of RT dose was based on tumor location, and elective nodal irradiation in the mediastinum may restrain adaptive immune responses^19^. When stratified for tumor location, patients with peripheral—but not centrally located—lesions showed increases in the proportion of both Ki67^+^CD8^+^ and Ki67^+^CD4^+^ circulating T-cells (Supplementary Fig. 2h, i).

**Fig. 2:**
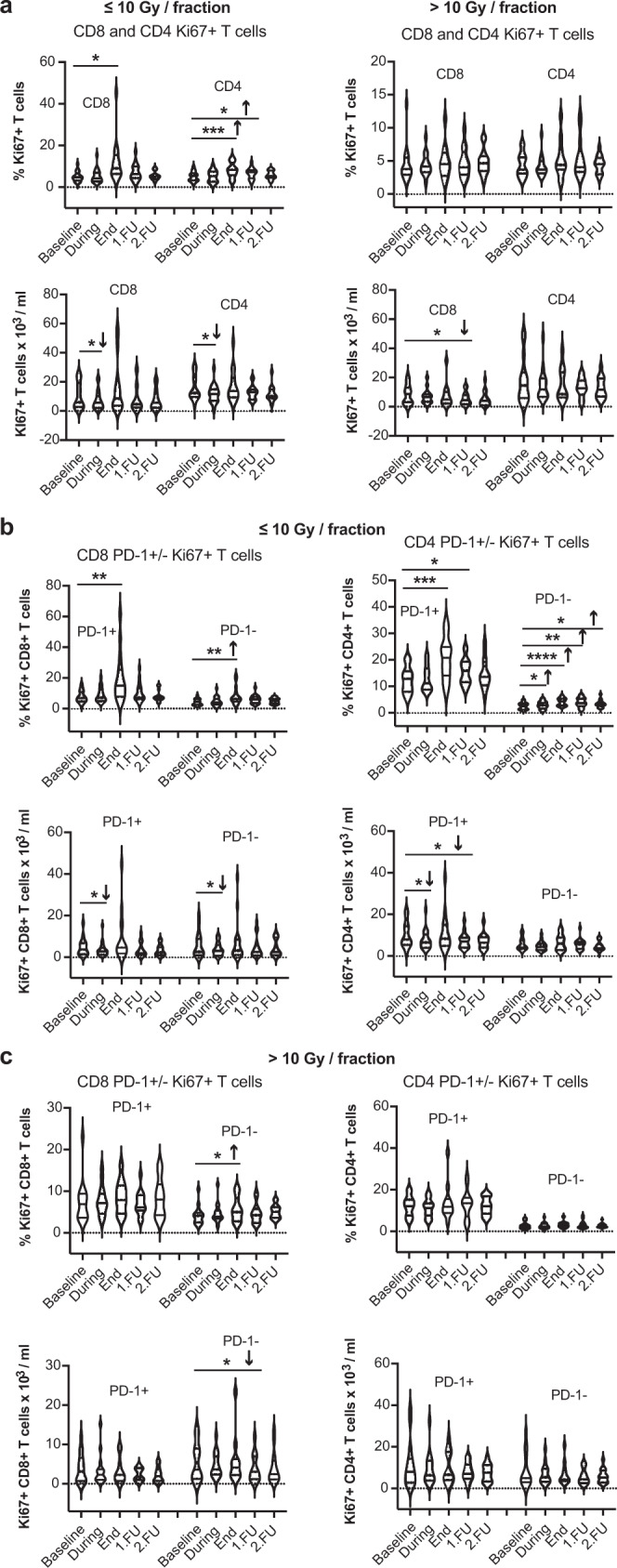
SBRT dose-dependent effects on CD8^+^ and CD4^+^ circulating T-cell proliferation post-treatment in early-stage NSCLC patients. **a** Fraction and absolute peripheral blood cell numbers of Ki-67^+^CD8^+^ and Ki-67^+^CD4^+^ T-cells after SBRT using doses of ≤10 Gy (left) versus >10 Gy (right). **b** Fraction and absolute peripheral blood cell numbers of Ki-67^+^PD-1^+^ versus Ki-67^+^PD-1^−^ CD8^+^ and CD4^+^ T-cells after SBRT using doses of ≤10 Gy. **c** Fraction and absolute peripheral blood cell numbers of Ki-67^+^PD-1^+^/ Ki-67^+^PD-1^−^ CD8^+^ and CD4^+^ T-cells with SBRT using doses >10 Gy. *p ≤ 0.05, **p < 0.01, ***p < 0.001, ****p < 0.0001 from mixed effects model for repeated measures with Geisser-Greenhouse correction and Benjamini, Krieger, and Yekutieli for the false discovery rate, two-sided. Arrows indicate the direction of change. Data are shown as median values (center lines) and interquartile ranges.

With a median follow-up of 31 months, median overall survival (OS) was not reached in this cohort, and the median progression-free survival (PFS) was 36 months (Supplementary Fig. 5a, b). At 2 and 4 years, the OS rates were 75% and 51%, respectively, and PFS rates were 56% and 25%, respectively. One patient developed local progression with a regional and distant progression and received treatment (SBRT, chemotherapy, and ICB). Four patients developed distant metastases (8%) and later received RT or ICB, and three patients had regional and distant metastases (6%) and received chemotherapy or palliative care. Nine patients developed regional recurrence (8%) and received RT or ICB (Supplementary Table 2).

In exploratory analyses, we detected no correlation between the biological effective dose (BED) and OS (hazard ratio per Gy [HR] = 0.99, 95% CI: 0.98–1.01, *p* = 0.4) or PFS (HR = 0.99, 95% CI: 0.98–1.01, *p* = 0.2) or between median BED and OS (*p* = 0.6). Moreover, there was no difference in outcomes between patients treated with more than 3 fractions (≤10 Gy per fraction) versus those who received 3 fractions (i.e., >10 Gy per fraction) (OS: HR = 1.50, 95% CI: 0.56–3.99, *p* = 0.4; PFS: HR = 1.60, 95% CI: 0.71–3.38, *p* = 0.3). Finally, we found that an increase in circulating CD8^+^ CTLs at FU1 (the primary study endpoint) was associated with a longer PFS (*p* = 0.043, log-rank test, Supplementary Fig. 5c); of note, the continuous ratio of CD8^+^ CTLs at FU1 compared to baseline showed a similar but statistically non-significant trend (HR = 0.29, 95% CI 0.67–1.308, *p* = 0.1). This result is hypothesis-generating and needs to be confirmed in larger studies.

These data show that SBRT can lead to transient lymphopenia in early-stage NSCLC, despite the smaller irradiated volumes. Interestingly, SBRT-induced lymphopenia was associated with increased T-cell proliferation, which may include tumor-specific T-cells^14,16,17,20^. Ablative SBRT could decrease the inhibitory signals from the tumors, reduce T-cell exhaustion and promote T-cell activation^21,22^. In patients with oligometastatic disease, SBRT has the potential to both reduce tumor burden and promote T-cell responses against micrometastases^6,22^. The increase in the expanding PD-1^+^CD8^+^ T-cell fraction usually peaks at 3–4 weeks after initiating ICB treatment^6,12^. In our study, the duration of SBRT was usually 1.5–2.5 weeks for patients treated with 5–8 fractions, and the expanding PD-1^+^CD8^+^ fraction peaked at the end of SBRT. The kinetics of these T-cell responses are potential biomarkers for optimally selecting patients and integrating ICB with SBRT in this setting.

The increased proliferation of CD8^+^ and CD4^+^ circulating T-cells in patients treated with 10 Gy or less per fraction could be due to immunogenic cancer cell death. Prior studies have suggested that moderate doses per fraction (8–12 Gy) induce cytoplasmic leakage of DNA and activation of cGAS/STING and primordial viral response pathways leading to the production of type-I IFN^8^. At higher doses, stimulation of DNA damage led to negative feedback expression of TREX1, which digested cytosolic DNA and reduced the IFN response and T-cell priming. These effects resulted in a lack of synergy with ICB^8^, in contrast to the enhanced efficacy of ICB when using the 3x8Gy dosing^23^.

This concept is currently tested in clinical trials, in which sub-ablative doses of 3×8 Gy of SBRT are combined with ICBs. One completed trial showed a significant prolongation of survival but did not meet the pre-specified endpoints^24^. Combining ICB with 3x8Gy sub-ablative SBRT correlated with increased activated TILs and circulating T-cells and decreased suppressive immune cells in head-and-neck squamous cell carcinomas^25^. Furthermore, multi-site SBRT with ICB (aPD1 + aCTLA4 antibodies) improved the intra-tumoral T-cell responses in patients with highly aneuploid metastatic NSCLCs^26^. Our results show that both reduction of tumor burden and systemic immune modulation are achieved only when using ablative SBRT with less than 10 Gy per fraction in single-site primary NSCLC. Studies of ICB with ablative SBRT with less than 10 Gy per fraction are warranted to confirm our findings and establish the timing, dose, and fractionation necessary to combine these modalities optimally.

Our study has limitations. Due to the different duration of SBRT regimens, post-treatment evaluations were not time matched. Since no patient received ICB combined with SBRT, future studies should determine whether and how combining SBRT with ICB impacts the changes in circulating T-cell proliferation in early-stage NSCLC.

In conclusion, our study shows that SBRT alone can significantly increase the fraction of proliferating CD4^+^ and CD8^+^ circulating T-cells, most prominently at the end of treatment and only in the cohort of early-stage NSCLC who received 10 Gy or less per fraction. These data might help decision-making for optimally integrating ICBs with SBRT in early-stage NSCLC and potentially other malignancies.

## Methods

The prospective study “LAPIS” was conducted in the Department of Radiation Oncology, University Medical Center Freiburg, Germany, and in the Department of Radiation Oncology, Massachusetts General Hospital (MGH) and Harvard Medical School Boston, USA, per the Declaration of Helsinki. The study was registered in the German trials registry (DRKS 00011266). All patients gave written informed consent according to institutional and federal guidelines. The institutional ethics committees approved the study protocol (EK 38/16, Freiburg and MGH IRB Agreement #:2016D009860). The prospective LA-PI-S trial enrolled patients with primary or recurrent non-metastatic lung cancer (*n* = 50) and liver cancer (*n* = 50) as well as patients with oligometastatic/oligoprogressive lung or liver metastases treated with SBRT without (lung *n* = 50, liver *n* = 50) or in combination with immunomodulating treatments (lung: *n* = 50, liver: *n* = 50). According to the protocol, each subgroup was analyzed separately. Herein we present the results of the circulating immune cell profiling of patients with early-stage non-small cell lung cancer (NSCLC) treated with SBRT by longitudinal assessment at first SBRT fraction (baseline), during and at the end of SBRT as well as at first (FU1) and second (FU2) follow up (6 weeks and another 3 months after the last SBRT fraction respectively). Patients with inoperable stage I–II judged suitable for SBRT by interdisciplinary consensus were enrolled in the study. All patients were previously staged with a ^18^F-FDG PET. Patients under systemic treatment, treatment with corticosteroids or other immunosuppressive drugs, and patients who received radiotherapy within the prior 3 months were deemed ineligible.

### Treatment planning and treatment delivery

Patients were immobilized in a supine position with a customized vacuum cushion system and received a 4D/CT or a 4D/PET-CT. Patients with peripheral tumors not abutting the chest wall received 3 × 18.75 Gy to the D50% such that 95% of the PTV received a minimum of 45 Gy (3x15Gy, 80% of the nominal dose) and a dose maximum between 110 and 120%^27^. Depending on the proximity to the central bronchial system and for tumors abutting or overlapping with the chest wall, a total dose of 50 Gy in 5 fractions of 10 Gy^28^ or 60 Gy in 8 fractions of 7.5 Gy^29^ for central tumors or 66 Gy in 12 fractions for ultra-central tumors. The dose prescription was chosen so that 95% of the PTV received at least the nominal fraction dose, and 99% of the PTV received a minimum of 90% of the nominal dose. The dose maximum within the PTV was chosen to be more than 110% but less than 120% of the prescribed dose. The aim was to apply a minimum biologically effective dose of 100 Gy. Central tumors were defined as tumors with one of the three following central chest locations: (1) within or touching the zone of the proximal bronchial tree, (2) within 5 mm or invading the mediastinal pleura, and (3) within 5 mm or invading the parietal pericardium^30^. The zone of the proximal bronchial tree was defined as per the Radiation Therapy Oncology Group (RTOG)^31^ as a volume of 2 cm in all directions around the carina, right and left main bronchi, right and left upper lobe bronchi, bronchus intermedius, right middle lobe bronchus, lingular bronchus, and right and left lower lobe bronchus.

Response to treatment was assessed at the same time points according to the Response Evaluation Criteria in Solid Tumors (RECIST) using thoracic CT and 18F-FDG PET/CT, the latter being mandatory in case of suspected disease progression.

Blood samples were collected by venipuncture before treatment (Baseline), 1 day after (During), at the end (End), at the 1st follow-up (FU1: 6 weeks after the end of SBRT), and the 2nd follow-up (FU2: 3 months after FU1). PBMCs were isolated and frozen until use. Samples were available from 27–42 patients at each time point. The reasons for the missing samples was that there were either not collected or had insufficient cells for all analyses. All results are summarized in Supplementary Table 3.

### Flow cytometry

Cryopreserved PBMC samples were thawed and washed in RPMI 1640 media. Samples were then resuspended in RPMI 1640 media and filtrated through a 30 µm preseparation filter (Miltenyi Biotec). Cells were counted, and live death staining was done using Zombie Red Fixable Viability stain (BioLegend), according to the manufacturer’s instructions. To detect surface markers, cells were incubated with a mixture of antibodies at 20´at 4 °C. All antibodies were used at a dilution of 1:200. Samples were fixed and permeabilized to detect intracellular antigens using the FoxP3 Fixation/Permeabilisation Kit from eBioscience. For in vitro restimulation of PBMCs to assess cytokine production, 10^6^ cells/ml were incubated in RPMI 1640 media with PMA (50 ng/ml), Ionomycin (1 μg/ml), and BFA (1:1000) for 5 h. After that, cells were stained for Zombie Red (BioLegend) for cell death exclusion and surface markers. Cells were then fixed with IC Fixation buffer (eBioscience) and stained for intracellular markers at 30´ at room temperature. Cells were stained in four different multicolor panels: MDSCs: HLA-DR-AF700 (L243, cat. no.307626), CD11b-PE (ICRF44, cat. no. 301306), CD33-APC (P67.6, cat. no. 366606); cytokines: CD3-FITC (OKT3, cat. no. 317306), CD4-BV510 (OKT4, cat. no. 317444), CD8-APC (HIT8a, cat. no. 300912), IFNγ−BV421 (B27, cat. no. 506538), IL-17A-PE (BL168, cat. no. 512306); Treg and activation markers: CD3-FITC, CD8-APC, CD4-BV510, CD25-PE-Cy7 (BC96, cat. no. 302612), CD127-PE (A019D5, cat. no. 351304), ICOS-PerCP-Cy5.5 (C398.4, cat. no. 313518), FoxP3-BV421 (206D, cat. no. 320124); T-cell proliferation and exhaustion markers: CD3-FITC, CD4-BV510, CD8-APC, PD-1-PE-Cy7 (EH12.1, cat. no. 561272), Tim3-PE (F38-2E2, cat. no. 345006), CTLA-4-BV605 (BNI3, cat. no. 369610), CD45RA-PerCP-Cy5.5 (HI100, cat. no. 304122), CCR7-AF700 (G043H7, cat. no. 353243), Ki67-BV421 (Ki-67, cat. no. 350506). All antibodies were purchased from BioLegend except PD-1-PE-Cy7, obtained from BD Biosciences. Analysis was performed on a Cytoflex S flow cytometer (Beckman Coulter). The online-only information shows the gating strategy (Supplementary Figs. 6 and 7).

### Statistical analysis

The study planned to include *n* = 50 patients with NSCLC (reported here), with pulmonary metastases, primary liver cancer, and hepatic metastases, respectively. This target sample size was derived based on feasibility considerations and the following considerations of statistical power. For the primary endpoint, the null hypothesis was that the probability of an increase (yes/no) of CD8^+^ counts 6 weeks after treatment compared to baseline, *p* (increase), is less than or equal to 50% (50% corresponds to no change from baseline to 6 weeks and a median post: pre CD8^+^ count ratio = 1, lower percentages correspond to a decrease and a median post versus pre CD8^+^ count ratio <1). The alternative hypothesis was *p* (increase) >50%. According to STPLAN (Version 4.5), an exact one-sided binomial test at a significance level of 5% would have at least 80% power to reject the null hypothesis if the true *p* (increase) is 68.5% or greater. The null hypothesis would be rejected if at least 32 out of 50 patients experienced an increase. The exact significance level is 3.25% (STPLAN version 4.5).

Intraindividual dynamic changes in blood biomarkers were examined using a mixed effects model for repeated measures with a Geisser-Greenhouse correction at four post-baseline time points compared to baseline (Supplementary Table 3). We performed a correction for multiple comparisons by controlling for a False Discovery Rate of 5% using a two-stage step method of Benjamini, Krieger, and Yekutiely within variables over time.

Overall survival (OS) and progression-free survival (PFS) were calculated from the start of SBRT and estimated according to the Kaplan–Meier method. For event-free patients, the observations for OS were censored at the date of the last contact and for PFS at the time of the last imaging or death. No patients died between baseline and end of treatment.

To investigate the correlation between parameters significantly changed at FU1 or FU2 compared to baseline, PFS was calculated from FU1 or FU2 in landmark analyses in the patients still at risk (alive and without progression at FU1 and FU2, respectively), respectively.

The impact of dose and fractionation on OS and PFS was estimated using a Cox regression. The correlation between changes in blood biomarkers and PFS was investigated using a log-rank test and Cox regression.

Differences were considered statistically significant when *p* values were less than 0.05. All *p* values are two-sided. Statistical analyses were performed using Prism (Prism V.8, GraphPad Software) and SPSS (IBM, SPSS, v27).

### Reporting summary

Further information on research design is available in the Nature Research Reporting Summary linked to this article.

## Supplementary information

Supplementary Information

REPORTING SUMMARY

## Data Availability

All data are included in this published article, and its Supplementary Information files and raw data are available from the corresponding author upon reasonable request to respect patient confidentiality.
