# Supplementary material for: Systemic immune modulation by stereotactic radiotherapy in early-stage lung cancer
Source: NPJ Precis Oncol. 2023 Mar 2;7:24. doi: 10.1038/s41698-023-00358-z (PMC9981559; doi:10.1038/s41698-023-00358-z)
Supplement: Supplementary file 1 — Supplementary Information [file 41698_2023_358_MOESM1_ESM.pdf]

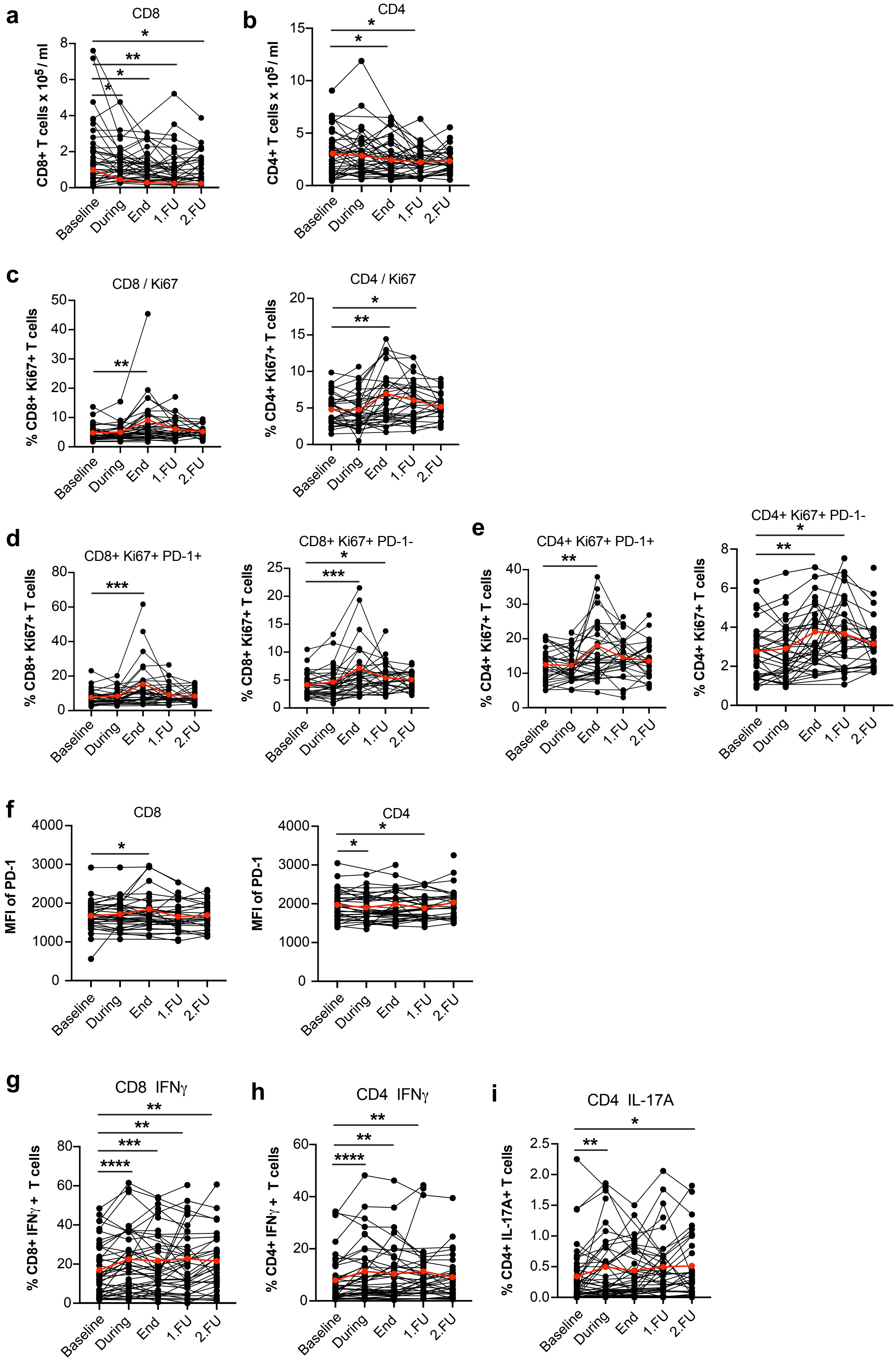
a
CD8
CD8+ T cells x 10^5 / ml
b
CD4
CD4+ T cells x 10^5 / ml
c
CD8 / Ki67
% CD8+ Ki67+ T cells
CD4 / Ki67
% CD4+ Ki67+ T cells
d
CD8+ Ki67+ PD-1+
% CD8+ Ki67+ T cells
CD8+ Ki67+ PD-1-
% CD8+ Ki67+ T cells
e
CD4+ Ki67+ PD-1+
% CD4+ Ki67+ T cells
CD4+ Ki67+ PD-1-
% CD4+ Ki67+ T cells
f
CD8
MFI of PD-1
CD4
MFI of PD-1
g
CD8 IFNγ
% CD8+ IFNγ + T cells
h
CD4 IFNγ
% CD4+ IFNγ + T cells
i
CD4 IL-17A
% CD4+ IL-17A+ T cells
Baseline
During
End
1.FU
2.FU

Supplementary Figure 1. Transient lymphodepletion during SBRT and increased proliferation of CD8$^+$ and CD4$^+$ circulating T-cells after SBRT in early-stage NSCLC patients. Data from combined SBRT schedules are shown as single curves for each patient and the mean values (in red.) (a) Absolute cell numbers of CD8$^+$ T-cells. **(b)** Absolute cell numbers of CD4$^+$ T-cells. **(c)** Percentage of Ki67$^+$CD8$^+$ and Ki67$^+$CD4$^+$ T-cells. **(d)** Percentage of PD-1$^+$ versus PD-1$^-$ Ki67$^+$CD8$^+$ T-cells. **(e)** Percentage of Ki67$^+$ PD-1$^+$ versus PD-1$^-$ CD4$^+$ T-cells. **(f)** Median fluorescence intensity (MFI) for PD-1 immunostaining on CD8$^+$ and CD4$^+$ T-cells. **(g)** Expression of IFN-γ in CD8$^+$ T-cells. **(h)** Expression of IFN-γ in CD4$^+$ T-cells. **(i)** Expression of IL-17A in CD4$^+$ T-cells. * $p<0.05$, ** $p<0.01$, *** $p<0.001$, **** $p<0.0001$ from mixed effects model for repeated measures with Geisser-Greenhouse correction and Benjamini, Krieger, and Yekutieli for the false Discovery Rate, two-sided.

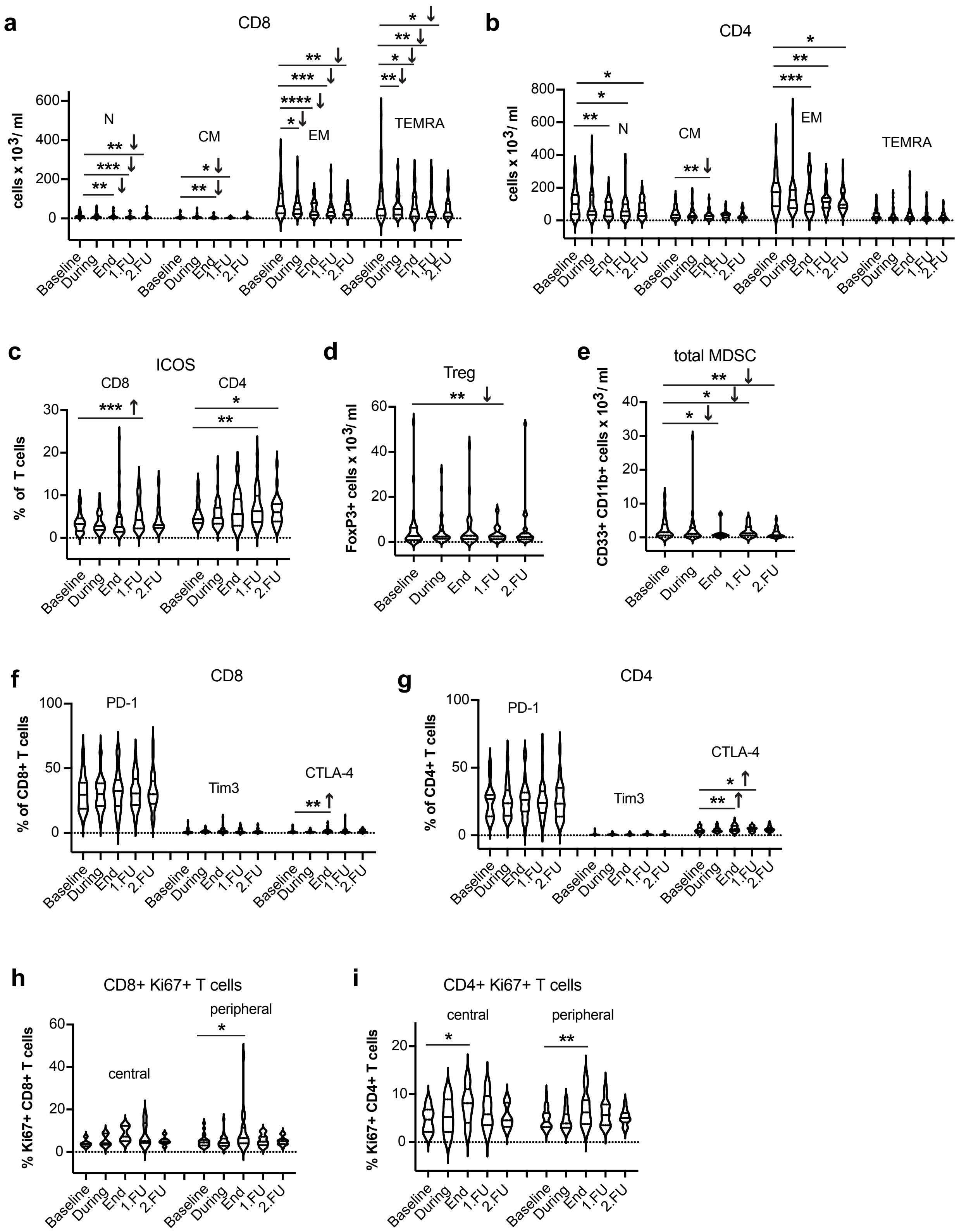
a
CD8
cells x 10^3/ ml
N
CM
EM
TEMRA
Baseline
During
End
1.FU
2.FU
b
CD4
cells x 10^3/ ml
N
CM
EM
TEMRA
c
ICOS
CD8
CD4
% of T cells
d
Treg
FoxP3+ cells x 10^3/ ml
e
total MDSC
CD33+ CD11b+ cells x 10^3/ ml
f
CD8
% of CD8+ T cells
PD-1
Tim3
CTLA-4
g
CD4
% of CD4+ T cells
PD-1
Tim3
CTLA-4
h
CD8+ Ki67+ T cells
% Ki67+ CD8+ T cells
central
peripheral
i
CD4+ Ki67+ T cells
% Ki67+ CD4+ T cells
central
peripheral

Supplementary Figure 2. SBRT causes a significant reduction of naïve and memory CD8$^+$ and CD4$^+$ circulating T-cell subpopulations, regulatory T cells (Treg), and myeloid-derived suppressor cells (MDSC) in early-stage NSCLC patients. (**Data presented include all SBRT schedules**.) **(a)** Absolute cell counts for naïve and memory CD8$^+$ T-cell subpopulations. **(b)** Absolute cell counts for naïve and memory CD4$^+$ T-cell subpopulations. **(c)** Fraction of ICOS$^+$ CD8$^+$ and CD4$^+$ T-cells. **(d)** Absolute Treg counts. **(e)** Absolute MDSC counts. **(f)** Fraction of PD-1$^+$, Tim3$^+$ and CTLA-4$^+$ CD8$^+$ T-cells. **(g)** Fraction of PD-1$^+$, Tim3$^+$ and CTLA-4$^+$ CD4$^+$ T-cells. Naïve and memory CD8$^+$ and CD4$^+$ T-cell subpopulations were defined as follows: naïve (N): CCR7$^+$CD45RA$^+$; central memory (CM): CCR7$^+$CD45RA$^-$; effector memory (EM): CCR7$^-$CD45RA$^-$; terminally differentiated effector memory re-expressing CD45RA (TEMRA): CCR7$^-$CD45RA$^+$. **(h, i)** Fraction of Ki67$^+$CD8$^+$ (**h**) Ki67$^+$CD4$^+$ (**i**) T-cells stratified by central versus peripheral tumor location. * $p<0.05$, ** $p<0.01$, *** $p<0.001$, **** $p<0.0001$ from mixed effects model for repeated measures with Geisser-Greenhouse correction and Benjamini, Krieger, and Yekutieli for the false Discovery Rate, two-sided. Arrows indicate the direction of change. Data are shown as median values (center lines) and interquartile ranges.

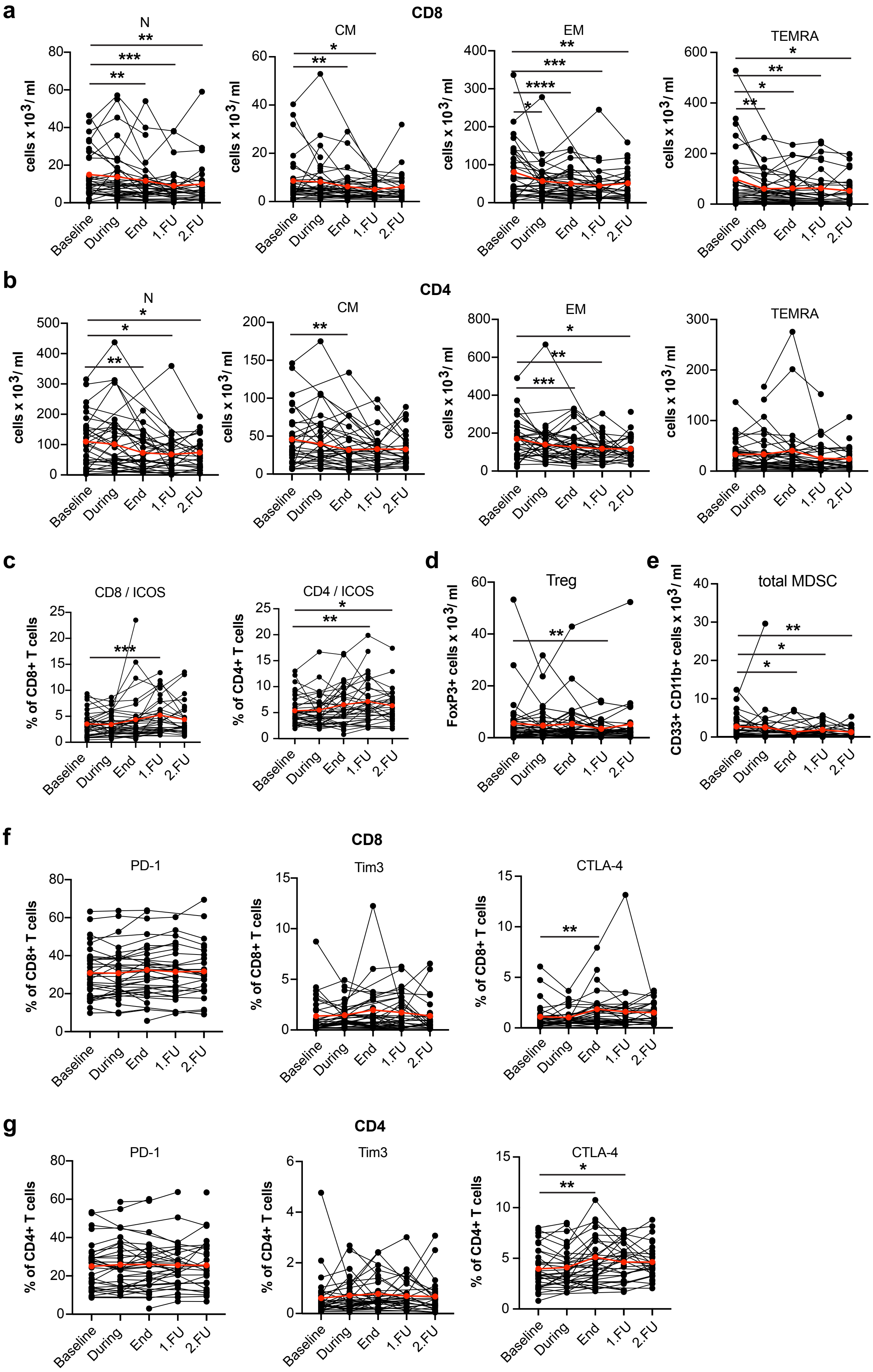

a
CD8
N
CM
EM
TEMRA
cells x 10³/ ml
Baseline
During
End
1.FU
2.FU
b
CD4
N
CM
EM
TEMRA
cells x 10³/ ml
c
CD8 / ICOS
% of CD8+ T cells
CD4 / ICOS
% of CD4+ T cells
d
Treg
FoxP3+ cells x 10³/ ml
e
total MDSC
CD33+ CD11b+ cells x 10³/ ml
f
CD8
PD-1
Tim3
CTLA-4
% of CD8+ T cells
g
CD4
PD-1
Tim3
CTLA-4
% of CD4+ T cells

Supplementary Figure 3. SBRT causes a significant reduction of naïve and memory CD8$^+$ and CD4$^+$ circulating T-cell subpopulations and inhibitory regulatory T cells (Treg) and myeloid-derived suppressor cells (MDSC) in NSCLC patients. (Data are from combined SBRT schedules, and are shown as individual changes each patient and mean values in red). (a) Absolute cell numbers of naïve and memory CD8$^+$ T-cell subpopulations. **(b)** Absolute cell numbers of naïve and memory CD4$^+$ T-cell subpopulations. **(c)** Percentage of ICOS$^+$ CD8$^+$ and CD4$^+$ T-cells. **(d)** Absolute cell numbers of Tregs. **(e)** Absolute cell numbers of MDSCs. **(f)** Percentage of PD-1$^+$, Tim3$^+$ and CTLA-4$^+$ CD8$^+$ T-cells. **(g)** Percentage of PD-1$^+$, Tim3$^+$ and CTLA-4$^+$ CD4$^+$ T-cells. Naïve and memory CD8$^+$ and CD4$^+$ T-cell subpopulations were defined as follows: naïve (N): CCR7$^+$CD45RA$^+$; central memory (CM): CCR7$^+$CD45RA$^-$; effector memory (EM): CCR7$^-$CD45RA$^-$; terminally differentiated effector memory re-expressing CD45RA (TEMRA): CCR7$^-$CD45RA$^+$. * $p<0.05$, ** $p<0.01$, *** $p<0.001$, **** $p<0.0001$ from mixed effects model for repeated measures with Geisser-Greenhouse correction and Benjamini, Krieger, and Yekutieli for the false Discovery Rate, two-sided.

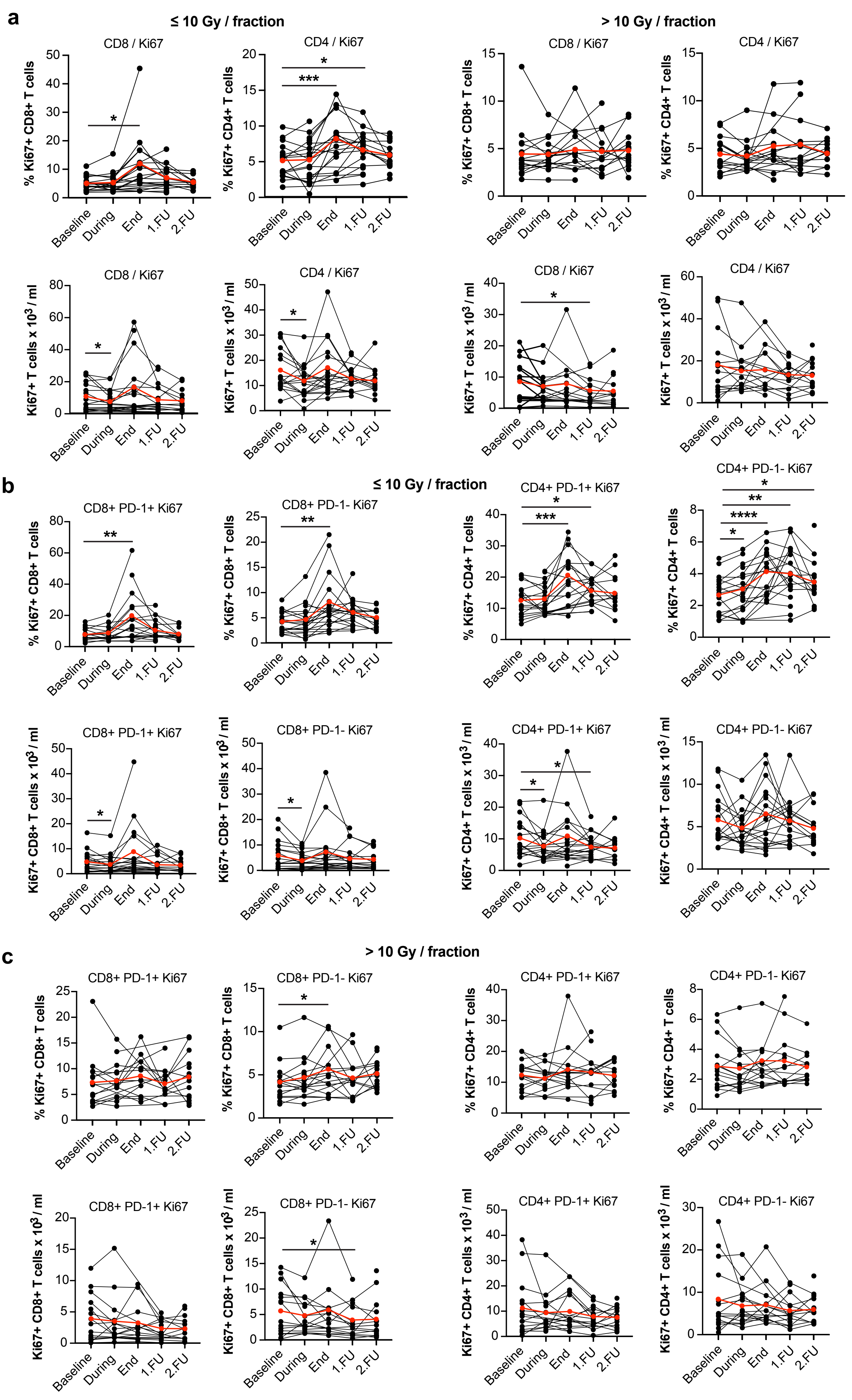
a
≤ 10 Gy / fraction
> 10 Gy / fraction
CD8 / Ki67
CD4 / Ki67
% Ki67+ CD8+ T cells
% Ki67+ CD4+ T cells
Ki67+ T cells x 10³ / ml
Baseline
During
End
1.FU
2.FU
b
≤ 10 Gy / fraction
CD8+ PD-1+ Ki67
CD8+ PD-1- Ki67
CD4+ PD-1+ Ki67
CD4+ PD-1- Ki67
% Ki67+ CD8+ T cells
% Ki67+ CD4+ T cells
Ki67+ CD8+ T cells x 10³ / ml
Ki67+ CD4+ T cells x 10³ / ml
c
> 10 Gy / fraction
CD8+ PD-1+ Ki67
CD8+ PD-1- Ki67
CD4+ PD-1+ Ki67
CD4+ PD-1- Ki67
% Ki67+ CD8+ T cells
% Ki67+ CD4+ T cells
Ki67+ CD8+ T cells x 10³ / ml
Ki67+ CD4+ T cells x 10³ / ml

Supplementary Figure 4. SBRT dose-dependent effects on CD8$^+$ and CD4$^+$ circulating T-cell proliferation post-treatment in early-stage NSCLC patients. Data are shown as curves for each patient and the mean values (in red). **(a)** Percentage and absolute cell numbers of Ki67$^+$CD8$^+$ and Ki67$^+$CD4$^+$ T-cells after SBRT using doses ≤ 10Gy (left) versus > 10Gy (right) per fraction. **(b)** Percentage and absolute cell numbers of PD-1$^+$ versus PD-1$^-$ Ki67$^+$CD8$^+$ and Ki67$^+$CD4$^+$ T-cells after SBRT using doses ≤ 10Gy per fraction. **(c)** Percentage and absolute cell numbers of PD-1$^+$ versus PD-1$^-$ Ki67$^+$CD8$^+$ and Ki67$^+$CD4$^+$ T-cells after SBRT using doses > 10Gy per fraction. * $p<0.05$, ** $p<0.01$, *** $p<0.001$, **** $p<0.0001$ from mixed effects model for repeated measures with Geisser-Greenhouse correction and Benjamini, Krieger, and Yekutieli for the false Discovery Rate, two-sided.

a. Overall survival

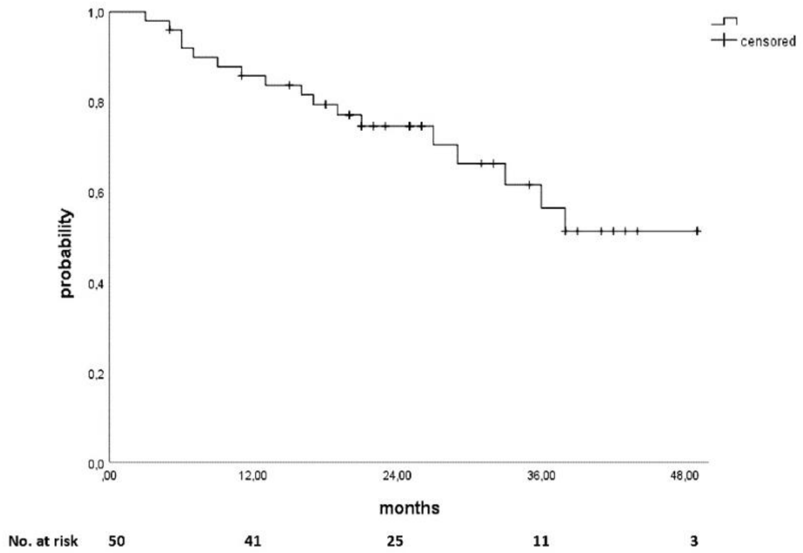

b. Progression free survival

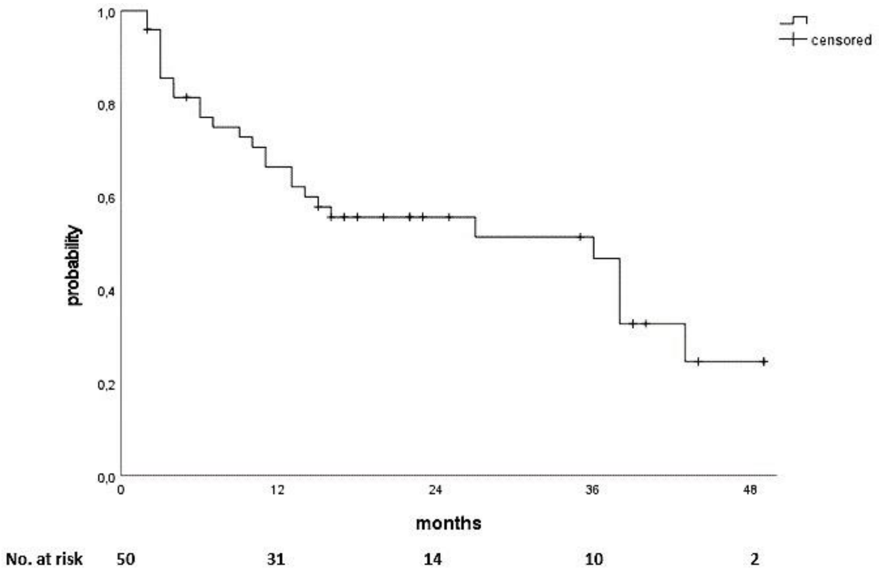

c. Progression free survival stratified according to CD 8+ T cell increase at first follow-up

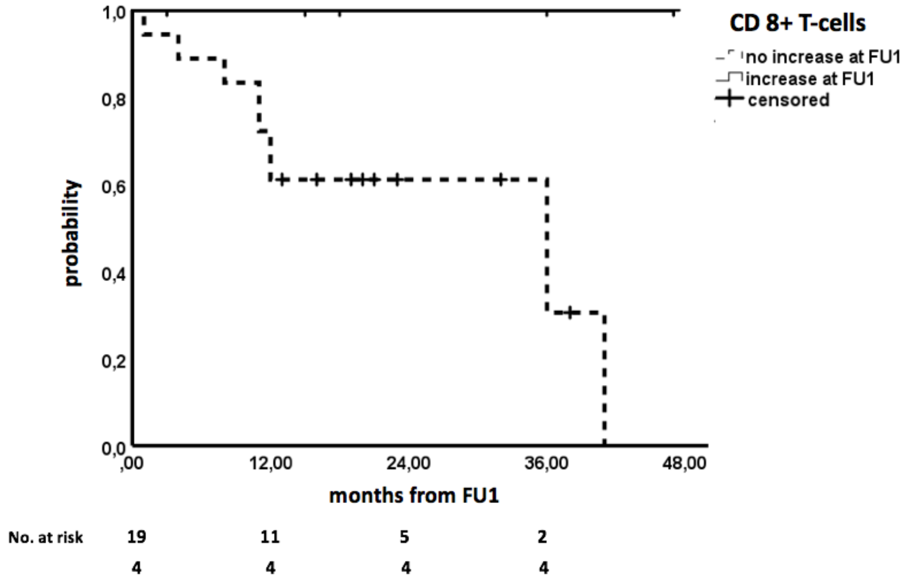

Supplementary Figure 5. Survival outcomes in LAPIS study. (a, b) Overall survival **(a)** and progression-free survival **(b)** in patients treated with SBRT were calculated from the start of SBRT (months). **(c)** Progression-free survival distributions from FU1 onwards in patients stratified by $CD8^+$ T-cell increase/no increase at FU1, the primary endpoint of this study (dashed lines). The number at risk is the number of patients who have neither been censored nor experienced progression or death.

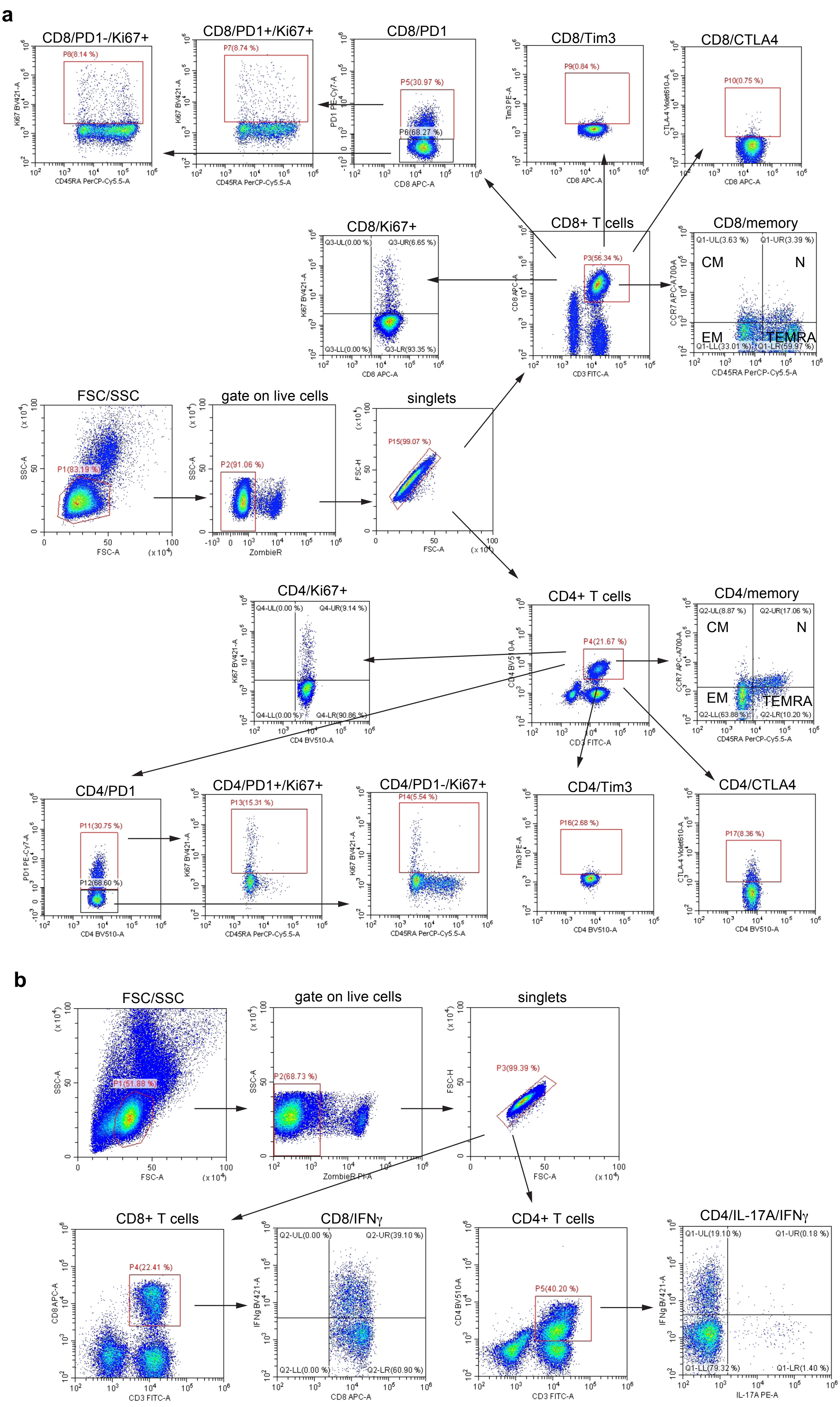
a
CD8/PD1-/Ki67+
P8(8.14 %)
CD8/PD1+/Ki67+
P7(8.74 %)
CD8/PD1
P5(30.97 %)
P6(68.27 %)
CD8/Tim3
P9(0.84 %)
CD8/CTLA4
P10(0.75 %)
CD8/Ki67+
Q3-UL(0.00 %)
Q3-UR(6.65 %)
Q3-LL(0.00 %)
Q3-LR(93.35 %)
CD8+ T cells
P3(56.34 %)
CD8/memory
Q1-UL(3.63 %)
Q1-UR(3.39 %)
Q1-LL(33.01 %)
Q1-LR(59.97 %)
CM
N
EM
TEMRA
FSC/SSC
P1(83.19 %)
gate on live cells
P2(91.06 %)
singlets
P15(99.07 %)
CD4/Ki67+
Q4-UL(0.00 %)
Q4-UR(9.14 %)
Q4-LL(0.00 %)
Q4-LR(90.86 %)
CD4+ T cells
P4(21.67 %)
CD4/memory
Q2-UL(8.87 %)
Q2-UR(17.06 %)
Q2-LL(63.88 %)
Q2-LR(10.20 %)
CM
N
EM
TEMRA
CD4/PD1
P11(30.75 %)
P12(68.60 %)
CD4/PD1+/Ki67+
P13(15.31 %)
CD4/PD1-/Ki67+
P14(5.54 %)
CD4/Tim3
P16(2.68 %)
CD4/CTLA4
P17(8.36 %)
Ki67 BV421-A
CD45RA PerCP-Cy5.5-A
PD1 PE-Cy7-A
CD8 APC-A
Tim3 PE-A
CTLA-4 Violet610-A
CCR7 APC-A700-A
CD3 FITC-A
SSC-A
FSC-A
FSC-H
ZombieR
CD4 BV510-A
b
FSC/SSC
P1(51.88 %)
gate on live cells
P2(68.73 %)
singlets
P3(99.39 %)
ZombieR PFA
CD8+ T cells
P4(22.41 %)
CD8/IFNγ
Q2-UL(0.00 %)
Q2-UR(39.10 %)
Q2-LL(0.00 %)
Q2-LR(60.90 %)
CD4+ T cells
P5(40.20 %)
CD4/IL-17A/IFNγ
Q1-UL(19.10 %)
Q1-UR(0.18 %)
Q1-LL(79.32 %)
Q1-LR(1.40 %)
IFNg BV421-A
IL-17A PE-A

Supplementary Figure 6. Gating strategy used to analyze peripheral blood immune cell subsets in early-stage NSCLC patients by flow cytometry. (a) Immunostaining panel to detect T cell proliferation, naïve and memory T cells, and T cell exhaustion: CD3-FITC, CD4-BV510, CD8-APC, PD-1-PE-Cy7, Tim3-PE, CTLA-4-BV605, CD45RA-PerCP-Cy5.5, CCR7-AF700, Ki67-BV421, and the live/dead marker Zombie Red. **(b)** Immunostaining panel to detect activation cytokines using the following marker: CD3-FITC, CD4-BV510, CD8-APC, IFNγ-BV421, IL-17A-PE, and the live/dead marker Zombie Red.

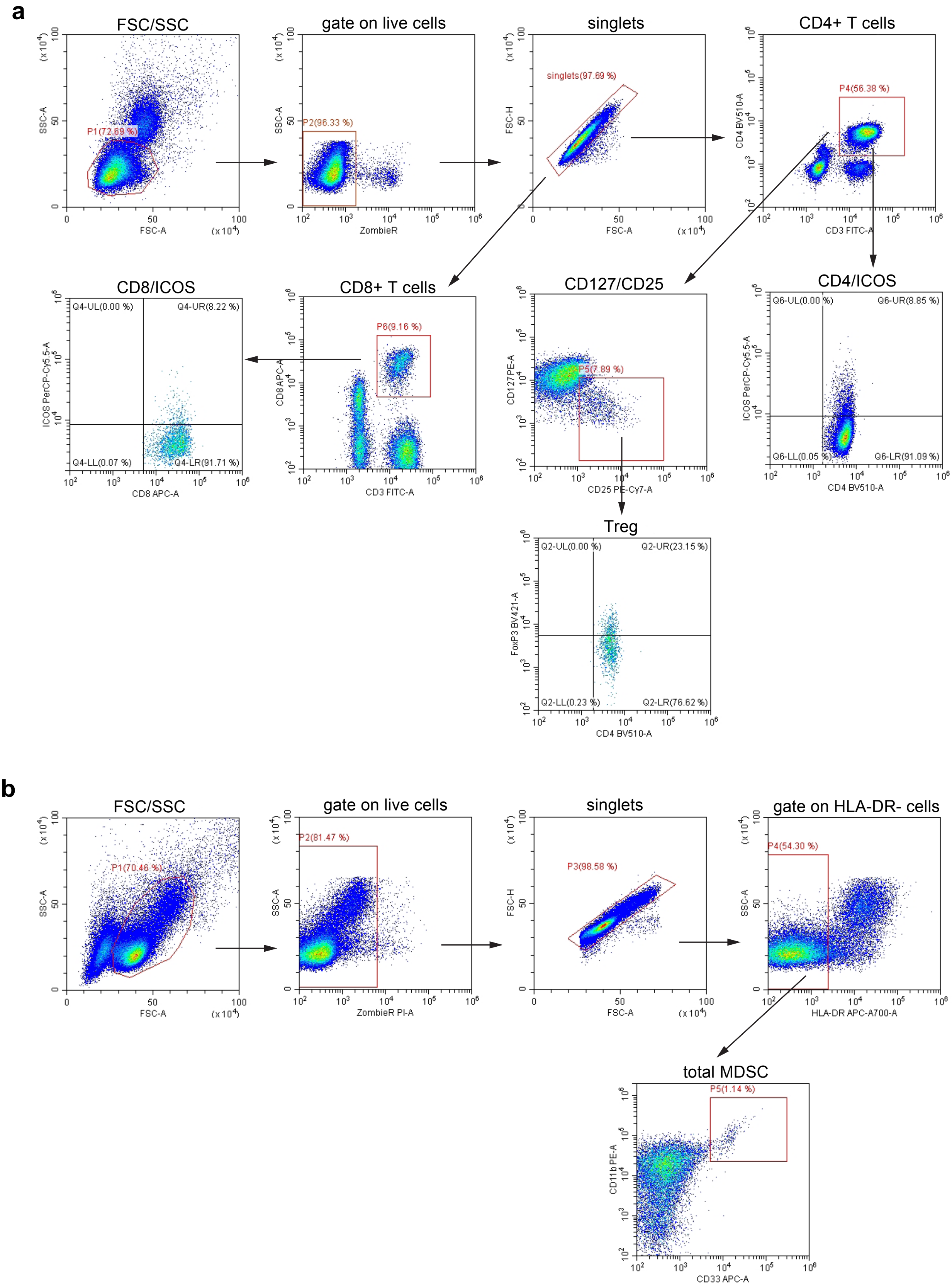
a
FSC/SSC
P1(72.69 %)
SSC-A
FSC-A
gate on live cells
P2(96.33 %)
ZombieR
singlets
singlets(97.69 %)
FSC-H
CD4+ T cells
P4(56.38 %)
CD4 BV510-A
CD3 FITC-A
CD8/ICOS
Q4-UL(0.00 %)
Q4-UR(8.22 %)
Q4-LL(0.07 %)
Q4-LR(91.71 %)
ICOS PerCP-Cy5.5-A
CD8 APC-A
CD8+ T cells
P6(9.16 %)
CD127/CD25
P5(7.89 %)
CD127 PE-A
CD25 PE-Cy7-A
CD4/ICOS
Q6-UL(0.00 %)
Q6-UR(8.85 %)
Q6-LL(0.05 %)
Q6-LR(91.09 %)
Treg
Q2-UL(0.00 %)
Q2-UR(23.15 %)
Q2-LL(0.23 %)
Q2-LR(76.62 %)
FoxP3 BV421-A
CD4 BV510-A
b
FSC/SSC
P1(70.46 %)
gate on live cells
P2(81.47 %)
ZombieR PI-A
singlets
P3(98.58 %)
gate on HLA-DR- cells
P4(54.30 %)
HLA-DR APC-A700-A
total MDSC
P5(1.14 %)
CD11b PE-A
CD33 APC-A

Supplementary Figure 7. Gating strategy used to analyze peripheral blood immune cell subsets in early-stage NSCLC patients by flow cytometry. **(a)** Immunostaining panel to detect regulatory T cells (Tregs) and activation markers: CD3-FITC, CD8-APC, CD4-BV510, CD25-PE-Cy7, CD127-PE, ICOS-PerCP-Cy5.5, FoxP3-BV421, and the live/dead marker Zombie Red. **(b)** Immunostaining panel to detect myeloid-derived suppressor cells (MDSCs): HLA-DR-AF700, CD11b-PE, CD33-APC, and the live/dead marker Zombie Red.

Supplementary Table 1. Patient and treatment characteristics

	Median (range) and number of patients (%)
Age (years)	70 (54-84)
Male<br>Female	34 (68%)<br>16 (32%)
Tumor localization<br>Left upper lobe<br>Left lower lobe<br>Right upper lobe<br>Right lower lobe	 13 (26%)<br>8 (16%)<br>19 (38%)<br>10 (20%)
Peripheral not abutting chest wall<br>Peripheral abutting/ overlapping chest wall<br>Central*<br>Ultra central	22 (44%)<br>14 (28%)<br>14 (28%)<br>1 (2%)
T N M<br>pTis N0 M0<br>T1a N0 M0<br>T1b N0 M0<br>T1c N0 M0<br>T2a N0 M0<br>T2b N0 M0<br>T3 N0 M0	 1 (2%)<br>2 (4%)<br>16 (32%)<br>17 (34%)<br>9 (18%)<br>3 (6%)<br>2 (4%)
UICC<br>0<br>IA1<br>IA2<br>IA3<br>IB<br>IIA<br>IIB	 1 (2%)<br>2 (4%)<br>16 (32%)<br>17 (34%)<br>9 (18%)<br>3 (6%)<br>2 (4%)
Maximum diameter (mm)	21 (10-58)
FEV1 (lt)	1.3 (0.34-3.4)
COPD<br>yes<br>no	 37 (74%)<br>13 (26%)
Total dose (Gy)	56.25 (50-66)
Dose per fraction (Gy)	10 (18.75-5.5)
BED (Gy)	105 (100-162)
BED D98% (Gy)	107 (90-143)
BED Dmedian (Gy)	123 (111-168)
BED D2% (Gy)	134 (122-216)
Number of fractions<br>3<br>5	 22 (44%)<br>6 (12%)

8	21 (42%)
12	1 (2%)

UICC: *Union for International Cancer Control, COPD:* chronic obstructive pulmonary disease, FEV1: Forced Expiratory Pressure in 1 Second, SCC: squamous cell carcinoma, BED: biological effective dose D:dose

*According to the RTOG definition by Timmerman et al.

Supplementary Table 2. Types of progression and subsequent treatments after radiotherapy

Type of progression	Number of patients (%)	Subsequent treatments
local	0	
regional	9 (18%)	Radiotherapy, ICB
distant	4 (8%)	Radiotherapy, ICB
local and regional	0	
local and distant	0	
local, regional and distant	1 (2%)	SBRT, chemotherapy, ICB
regional and distant	3 (6%)	Chemotherapy, palliative care

ICB: immune checkpoint blockade

Supplementary Table 3. Changes in all circulating cell biomarkers compared to baseline

	Baseline		During			End			FU1			FU2		
Variable	n	mean	n*	Mean difference from baseline	p	n*	Mean difference from baseline	p	n*	Mean difference from baseline	p	n*	Mean difference from baseline	p
CD8+ T-cells	36	1.856	32	-0.4683	0.02	25	-0.6181	0.02	28	-0.6663	0.001	24	-0.6624	0.01
CD4+ T-cells	37	3.066	34	-0.2134	0.41	29	-0.6143	0.02	31	-0.8257	0.03	26	-0.7271	0.05
% CD4 INFγ T-cells	42	7.937	37	3.403	0.0001	33	2.415	0.005	33	3.390	0.005	26	1.160	0.28
%CD4 PD1+	32	25.03	28	0.8928	0.21	23	0.9693	0.36	24	0.5778	0.55	20	0.5389	0.65
%CD4 Tim3+	32	0.6116	28	0.1064	0.52	23	0.1729	0.65	24	0.08610	0.44	20	0.06522	0.77
%CD4 CTLA4+	32	3.959	28	0.1315	0.49	23	1.151	0.05	24	0.6619	0.03	20	0.6742	0.05
%CD4 ICOS	37	5.375	34	0.1535	0.65	25	1.175	0.07	29	1.795	0.002	25	0.9952	0.03
%CD4 IL17A T cells	42	0.3412	36	0.1531	0.009	32	0.0969	0.139	33	0.1531	0.07	27	0.1722	0.04
%CD4 KI67+ T cells	32	4.768	28	-0.01754	0.95	23	2.207	0.003	26	1.310	0.02	22	0.4004	0.23
%CD4 KI67+ PD1+	32	12.40	29	-0.2017	0.71	23	5.536	0.002	26	2.144	0.08	20	1.077	0.34
%CD4 KI67+ PD1-	32	2.756	29	0.1444	0.29	23	1.011	0.002	26	0.9031	0.01	20	0.3933	0.09
%CD8 INFγ T-cells	42	16.90	36	5.460	0.0001	32	4.683	0.0008	34	5.710	0.002	27	4.515	0.004
%CD8 PD1+	32	30.91	28	-0.1128	0.87	24	1.582	0.08	24	0.6086	0.38	20	0.7941	0.28
%CD8 Tim3+	32	1.363	28	0.09274	0.67	24	0.6080	0.18	24	0.3582	0.25	20	0.01978	0.95
%CD8 CTLA4+	32	1.098	28	-0.06403	0.68	24	0.7405	0.007	24	0.4952	0.13	20	0.4141	0.09
%CD8 KI67+ T-cells	32	4.780	28	0.1509	0.62	23	4.347	0.006	26	1.247	0.09	22	0.3878	0.57
%CD8 KI67+ PD1+	32	7.578	29	0.6919	0.16	23	7.677	0.0009	26	1.431	0.21	20	0.6401	0.56
%CD8 KI67+ PD1-	32	4.174	29	0.4645	0.07	23	2.954	0.0007	25	1.248	0.03	20	0.8934	0.08
%CD8 ICOS	37	3.528	34	-0.09611	0.71	25	0.8282	0.32	29	1.691	0.0004	25	0.8495	0.05
MFI of PD-1 CD8	32	1671	28	39.38	0.31	22	167.6	0.04	26	-23.05	0.66	20	12.74	0.81
MFI of PD-1 CD4	32	1972	28	-69.17	0.01	22	20.18	0.6435	26	-86.56	0.02	20	63.41	0.12
Treg	36	5.529	32	-0.8311	0.52	25	-0.1850	0.73	29	-2.090	0.004	24	-0.3728	0.65

MDSC	29	2.669	25	-0.2186	0.80	15	-1.373	0.02	23	-0.8626	0.05	23	-1.443	0.003
CD4 N	32	110.3	29	-8.657	0.40	23	-36.59	0.002	24	-42.29	0.02	21	-36.13	0.01
CD4 CM	32	45.95	29	-6.232	0.21	23	-14.00	0.007	24	-12.92	0.06	21	-13.27	0.06
CD4 EM	32	170.1	29	-29.91	0.07	23	-42.83	0.0009	24	-51.98	0.009	21	-53.13	0.02
CD4 TEMRA	32	32.70	29	0.3295	0.89	23	7.356	0.20	24	-7.700	0.10	21	-8.793	0.07
CD8 N	32	14.91	29	-1.236	0.44	23	-3.365	0.008	24	-5.942	0.0004	21	-5.049	0.008
CD8 CM	32	8.594	29	-0.4036	0.76	23	-2.375	0.006	24	-3.558	0.02	21	-2.406	0.11
CD8 EM	32	80.79	29	-23.75	0.03	23	-30.17	0.0001	24	-35.50	0.0002	21	-29.23	0.008
CD8 TEMRA	32	98.67	29	-37.90	0.001	23	-35.97	0.05	24	-35.73	0.009	21	-46.17	0.01

*number of patients with blood draws at baseline and the corresponding time point
