# Supplementary material for: Systemic immune modulation by stereotactic radiotherapy in early-stage lung cancer
Source: NPJ Precis Oncol. 2023 Mar 2;7:24. doi: 10.1038/s41698-023-00358-z (PMC9981559; doi:10.1038/s41698-023-00358-z)
Supplement: Supplementary file 2 — REPORTING SUMMARY [file 41698_2023_358_MOESM2_ESM.pdf]

nature research

Corresponding author(s): Dan G. Duda

Last updated by author(s): Jun 24, 2022

Reporting Summary

Nature Research wishes to improve the reproducibility of the work that we publish. This form provides structure for consistency and transparency in reporting. For further information on Nature Research policies, see our Editorial Policies and the Editorial Policy Checklist.

Statistics

For all statistical analyses, confirm that the following items are present in the figure legend, table legend, main text, or Methods section.

n/a	Confirmed	
☐	☒	The exact sample size (*n*) for each experimental group/condition, given as a discrete number and unit of measurement
☐	☒	A statement on whether measurements were taken from distinct samples or whether the same sample was measured repeatedly
☐	☒	The statistical test(s) used AND whether they are one- or two-sided<br>*Only common tests should be described solely by name; describe more complex techniques in the Methods section.*
☐	☒	A description of all covariates tested
☐	☒	A description of any assumptions or corrections, such as tests of normality and adjustment for multiple comparisons
☐	☒	A full description of the statistical parameters including central tendency (e.g. means) or other basic estimates (e.g. regression coefficient) AND variation (e.g. standard deviation) or associated estimates of uncertainty (e.g. confidence intervals)
☐	☒	For null hypothesis testing, the test statistic (e.g. *F*, *t*, *r*) with confidence intervals, effect sizes, degrees of freedom and *P* value noted<br>*Give P values as exact values whenever suitable.*
☒	☐	For Bayesian analysis, information on the choice of priors and Markov chain Monte Carlo settings
☒	☐	For hierarchical and complex designs, identification of the appropriate level for tests and full reporting of outcomes
☒	☐	Estimates of effect sizes (e.g. Cohen's *d*, Pearson's *r*), indicating how they were calculated

Our web collection on statistics for biologists contains articles on many of the points above.

Software and code

Policy information about availability of computer code

Data collection	FACS analysis: Samples were acquired by Cytoflex S (Beckman Coulter) flow cytometry.<br>Microsoft Excel: version 2016; Treatment planning: Eclipse version 15.1.
Data analysis	FACS data were analysed by Cytoflex S analysis software (Beckman Coulter)<br>Statistical analyses were performed using Prism (Prism V.8, GraphPad Software) and SPSS (IBM, SPSS, v27).

For manuscripts utilizing custom algorithms or software that are central to the research but not yet described in published literature, software must be made available to editors and reviewers. We strongly encourage code deposition in a community repository (e.g. GitHub). See the Nature Research guidelines for submitting code & software for further information.

Data

Policy information about availability of data

All manuscripts must include a data availability statement. This statement should provide the following information, where applicable:

- Accession codes, unique identifiers, or web links for publicly available datasets
- A list of figures that have associated raw data
- A description of any restrictions on data availability

All relevant data are included in the paper and in the supplementary information files.

Field-specific reporting

Please select the one below that is the best fit for your research. If you are not sure, read the appropriate sections before making your selection.

☒ Life sciences ☐ Behavioural & social sciences ☐ Ecological, evolutionary & environmental sciences

For a reference copy of the document with all sections, see nature.com/documents/nr-reporting-summary-flat.pdf

Life sciences study design

All studies must disclose on these points even when the disclosure is negative.

Sample size	This target sample size of was derived based on feasibility considerations and the following considerations of statistical power. For the primary endpoint, the null hypothesis was that the probability of an increase (yes/no) of CD8+ counts six weeks after treatment compared to baseline, P (increase), is less than or equal to 50% (50% corresponds to no change from baseline to six weeks and to a median post:pre CD8+ count ratio = 1, lower percentages correspond to a decrease and to a median post:pre CD8+ count ratio < 1). The alternative hypothesis was P (increase) > 50%. According to STPLAN (Version 4.5), an exact one-sided binomial test at significance level 5% would have at least 80% power to reject the null hypothesis if the true P(increase) is 68.5% or greater. The null hypothesis would be rejected if at least 32 out of 50 patients experienced an increase. The exact significance level is 3.25% (STPLAN version 4.5).The sample size is provided in the online materials and methods section.
Data exclusions	No data were excluded.
Replication	No replication was performed due to insufficient cell numbers per sample.
Randomization	No randomization was performed.
Blinding	Experiments were generally blinded.

Reporting for specific materials, systems and methods

We require information from authors about some types of materials, experimental systems and methods used in many studies. Here, indicate whether each material, system or method listed is relevant to your study. If you are not sure if a list item applies to your research, read the appropriate section before selecting a response.

Materials & experimental systems

n/a	Involved in the study	
☐	☒	Antibodies
☒	☐	Eukaryotic cell lines
☒	☐	Palaeontology and archaeology
☒	☐	Animals and other organisms
☐	☒	Human research participants
☐	☒	Clinical data
☒	☐	Dual use research of concern

Methods

n/a	Involved in the study	
☒	☐	ChIP-seq
☐	☒	Flow cytometry
☒	☐	MRI-based neuroimaging

Antibodies

Antibodies used	All antibodies (name, vendor, clone, dilution factor) were provided in the method section (under Flow cytometry). The following antibodies were used: HLA-DR (Biolegend, cat. no. 307626, clone L243), CD11b (Biolegend, cat. no. 301306, clone ICRF44), CD33 (Biolegend, cat. no. 366606, clone P67.6), CD3 (Biolegend, cat. no. 317306, clone OKT3), CD4 (Biolegend, cat. no. 317444, clone OKT4), CD8 (Biolegend, cat. no. 300912, clone HIT8a), IFNg (Biolegend, cat. no. 506538, clone B27), IL17A (Biolegend, cat. no. 512306, clone BL168), CD25 (Biolegend, cat. no. 302612, clone BC96), CD127 (Biolegend, cat. no. 351304, clone A019D5), ICOS (Biolegend, cat. no. 313518, clone C398.4), FoxP3 (Biolegend, cat. no. 320124, clone 206D), PD-1 (BD Biosciences, cat. no. 561272, clone EH12.1), CTLA-4 (Biolegend, cat. no. 369610, clone BNI3), CD45RA (Biolegend, cat. no. 304122, clone HI100), CCR7 (Biolegend, cat. no. 353243, clone G043H7), Ki-67 (Biolegend, cat. no. 350506, clone Ki67).
Validation	All primary antibodies were bought from vendors (Biolegend and BD Biosciences) and used for the specifies suggested by the manufacturers. All antibodies were validated by the suppliers. Validations can be found in the manufacturer´s website. Many of the antibodies are routinely used antibodies. Flow data suggests clear separation of the cell populations.

Human research participants

Policy information about studies involving human research participants

Population characteristics	Patients (male 68%, female 32%) with inoperable stage I-II judged to be suitable for SBRT by interdisciplinary consensus were enrolled in the study. The median age was 70 years. All patients were previously staged with a ^{18}F-FDG PET. Patients under systemic treatment, treatment with corticosteroids or other immunosuppressive drugs as well as patients with previous radiotherapy within the last 3 months were deemed ineligible.Patients were immobilized in supine position with a customised vacuum cushion system and received a 4D/CT or a 4D/PET-CT. Patients with peripheral tumors not abutting the chest wall received 3 x 18.75Gy to the D50% such that 95% of the PTV received a minimum of 45 Gy (3x15Gy, 80% of the nominal dose) and a dose maximum between 110 and 120%. Depending on the proximity to the central bronchial system and for tumors abutting or overlapping with the chest wall, a total dose of 50Gy in 5 fractions of 10Gy or 60Gy in 8 fractions of 7.5Gy for central tumors or 66Gy in 12 fractions for ultra-central tumors. The dose prescription was chosen so that 95% of the PTV received at least the nominal fraction dose, and 99% of the PTV received a minimum of 90% of the nominal dose. The dose maximum within the PTV was chosen to be more than 110% but less than 120% of the prescribed dose. The aim was to apply a minimum biologically effective dose of 100Gy. Central tumors were defined as tumors with one of the three following central chest locations: (1) within or touching the zone of the proximal bronchial tree, (2) within 5 mm or invading the mediastinal pleura, and (3) within 5 mm or invading the parietal pericardium The zone of the proximal bronchial tree was defined as per the Radiation Therapy Oncology Group (RTOG) as a volume of 2 cm in all directions around the carina, right and left main bronchi, right and left upper lobe bronchi, bronchus intermedius, right middle lobe bronchus, lingular bronchus, and right and left lower lobe bronchus.<br>Response to treatment was assessed at the same time points according to the Response Evaluation Criteria in Solid Tumors (RECIST) using thoracic CT and 18F-FDG PET/CT, the latter being mandatory in case of suspected disease progression.<br>Blood samples were collected by venipuncture before treatment (Baseline), 1 day after (During), at the end (End), at the 1st follow-up (FU1: six weeks after the end of SBRT), and the 2nd follow-up (FU2: 3 months after FU1). PBMCs were isolated and frozen until use. Samples were available from 27-42 patients at each time point. The reasons for the missing samples was that there were either not collected or had insufficient cells for all analyses.
Recruitment	The prospective LA-PI-S trial enrolled patients with primary or recurrent non-metastatic lung cancer (n=50) and liver cancer (n=50) as well as patients with oligometastatic/oligoprogressive lung or liver metastases treated with SBRT without (lung n=50, liver n=50) or in combination with immunomodulating treatments (lung: n=50, liver: n=50). According to the protocol, each subgroup was analyzed separately. Herein we present the results of the circulating immune cell profiling of patients with early-stage non-small cell lung cancer (NSCLC) treated with SBRT by longitudinal assessment at first SBRT fraction (baseline), during and at the end of SBRT as well as at first (FU1) and second (FU2) follow up (six weeks and another 3 months after the last SBRT fraction respectively).
Ethics oversight	EK 38/16, Freiburg and MGH IRB Agreement #:2016D009860

Note that full information on the approval of the study protocol must also be provided in the manuscript.

Clinical data

Policy information about clinical studies

All manuscripts should comply with the ICMJE guidelines for publication of clinical research and a completed CONSORT checklist must be included with all submissions.

Clinical trial registration	The trial registration number is DRKS 00011266.
Study protocol	The study protocol can be accessed under EK 38/16, Freiburg and MGH IRB Agreement #: 2016D009860.
Data collection	All data were collected in the University Medical Center Freiburg, Dept. of Radiation Oncology. Response to treatment was assessed at the same time points according to the Response Evaluation Criteria in Solid Tumors (RECIST) by means of thoracic CT and/or 18F-FDG PET/CT, the latter being mandatory in case of suspected disease progression. Blood samples were collected by venepuncture prior treatment, 1 day after the first SBRT, at the end of SBRT, at the 1st follow-up (6 weeks after the end of SBRT) and at the 2nd follow-up (3 months after the 1st follow-up).
Outcomes	The pre-specified primary endpoint was increase (yes/no) in circulating CD8+ CTL counts at FU1 compared to pre-treatment, and secondary endpoints included changes in other T-cell subsets at all time-points.

Flow Cytometry

Plots

Confirm that:

☒ The axis labels state the marker and fluorochrome used (e.g. CD4-FITC).

☒ The axis scales are clearly visible. Include numbers along axes only for bottom left plot of group (a 'group' is an analysis of identical markers).

☒ All plots are contour plots with outliers or pseudocolor plots.

☒ A numerical value for number of cells or percentage (with statistics) is provided.

Methodology

Sample preparation	Patient blood samples were collected by venepuncture (BD Vacutainer, cat. no. 362782, BD). PBMCs were isolated by centrifugation of the blood collection tubes at 1650xg for 20 minutes. Isolated PBMCs were frozen until use.PBMC samples were thawed and washed in RPMI 1640 media. Samples were then resuspended in RPMI 1640 media and filtrated through a 30 µm preseparation filter (Miltenyi Biotec). Cells were then counted and live death staining was done using Zombie Red Fixable Viability stain (BioLegend), according to the manufacturer instructions. For detection of surface markers, cells were incubated with a mixture of antibodies for 20´at 4°C. For detection of intracellular antigens samples were fixed and permeabilized using the FoxP3 Fixation/Permeabilisation Kit from eBioscience. For in vitro restimulation of PBMCs 106 cells/ml were incubated in RPMI 1640 media with PMA (50 ng/ml), Ionomycin (1 g/ml) and BFA (1:1000) for 5h. Thereafter cells were stained for Zombie Red (BioLegend) for cell death exclusion and then surface markers. Cells were then fixed with IC Fixation buffer (eBioscience) and stained for intracellular markers, 30´at room temperature. Cells were stained in 4 different multicolor panels: MDSCs: HLA-DR-AF700 (L243), CD11b-PE (ICRF44), CD33-APC (P67.6); cytokines: CD3-FITC (OKT3), CD4-BV510 (OKT4), CD8-APC (HIT8a), IFN BV421 (B27), IL-17A-PE (BL168); Treg and activation markers: CD3-FITC, CD8-APC, CD4-BV510, CD25-PE-Cy7 (BC96), CD127-PE (A019D5), ICOS-PerCP-Cy5.5 (C398.4), FoxP3-BV421 (206D); T cell proliferation and exhaustion markers: CD3-FITC, CD4-BV510, CD8-APC, PD-1-PE-Cy7 (EH12.1), Tim3-PE (F38-2E2), CTLA-4-BV605 (BNI3), CD45RA-PerCP-Cy5.5 (HI100), CCR7-AF700 (G043H7), Ki67-BV421 (Ki-67). All antibodies were purchased from BioLegend except PD-1-PE-Cy7, which was obtained from BD Biosciences. Analysis was performed on a Cytoflex S flow cytometer (Beckman Coulter).
Instrument	Samples were analysed on a flow cytometer (Cytoflex S, Beckman Coulter).
Software	Data were analysed with the Cytoflex S analysis software.
Cell population abundance	The numbers presented in the flow cytometry images are percentage based. Only PBMCs were analysed.
Gating strategy	In the FSC-A/SSC-A, cells were gated for the relevant cell populations (lymphocytes or whole PBMCs). Cells were then gated for living cells, which are negative for the dead cell marker Zombie Red. Then cells were gated for singlets according to FSC-H/FSC-A. Then cell populations were determined according to the specific antibodies. Positive populations were clearly separated from negative cell populations.

☒ Tick this box to confirm that a figure exemplifying the gating strategy is provided in the Supplementary Information.
